# Candidate Loci for Yield-Related Traits in Maize Revealed by a Combination of MetaQTL Analysis and Regional Association Mapping

**DOI:** 10.3389/fpls.2017.02190

**Published:** 2017-12-22

**Authors:** Lin Chen, Yixin An, Yong-xiang Li, Chunhui Li, Yunsu Shi, Yanchun Song, Dengfeng Zhang, Tianyu Wang, Yu Li

**Affiliations:** Institute of Crop Sciences, National Key Facility for Crop Gene Resources and Genetic Improvement, Chinese Academy of Agricultural Sciences, Beijing, China

**Keywords:** grain yield, kernel size and weight, metaQTL, regional association mapping, maize

## Abstract

Maize grain yield and related traits are complex and are controlled by a large number of genes of small effect or quantitative trait loci (QTL). Over the years, a large number of yield-related QTLs have been identified in maize and deposited in public databases. However, integrating and re-analyzing these data and mining candidate loci for yield-related traits has become a major issue in maize. In this study, we collected information on QTLs conferring maize yield-related traits from 33 published studies. Then, 999 of these QTLs were iteratively projected and subjected to meta-analysis to obtain metaQTLs (MQTLs). A total of 76 MQTLs were found across the maize genome. Based on a comparative genomics strategy, several maize orthologs of rice yield-related genes were identified in these MQTL regions. Furthermore, three potential candidate genes (Gene ID: GRMZM2G359974, GRMZM2G301884, and GRMZM2G083894) associated with kernel size and weight within three MQTL regions were identified using regional association mapping, based on the results of the meta-analysis. This strategy, combining MQTL analysis and regional association mapping, is helpful for functional marker development and rapid identification of candidate genes or loci.

## Introduction

Maize is one of the most important cereal crops in the world and plays an important role in maintaining food security, promoting the development of animal husbandry, and satisfying the demand for industrial raw materials. Thus, improvement of grain yield is consistently one of the most important goals for maize breeders. In maize, grain yield is a complex quantitative trait controlled by many quantitative trait loci (QTL) with a small effect (Wu and Lin, 2006). A better understanding of the genetic architecture and molecular mechanisms of yield-related traits could help improve grain yield in maize.

Linkage mapping is an efficient way to identify genetic loci for complex quantitative traits in maize (Wallace et al., 2014). In the past two decades, many QTLs have been identified for yield and related traits thus far, such as ear-related traits (ERT) and kernel-related traits (http://www.maizegdb.org/). Marker-assisted selection (MAS) is a more efficient selection method for yield and its related traits improvement in the process of crop breeding. The tightly linked markers which are found in the genetic population and related with yield and its related traits should be identified before using in the MAS for crop breeding process (Xu, 2010). However, it is difficult to use these loci in the crop improvement process for the following reasons: (1) the results of QTL mapping for the same trait may vary due to the different populations used in different studies; (2) most QTLs explain just a small proportion of phenotypic variation and are detected only in specific environments; and (3) the confidence intervals for these QTLs are often large and contain hundreds of genes, making it very difficult to determine the candidate gene for the target trait.

QTL meta-analysis is an effective method to identify the genomic hotspot regions that control target traits more frequently and narrow down the confidence intervals of these QTLs to produce the metaQTLs (MQTLs) by integrating information from different mapping populations (Goffinet and Gerber, 2000; Arcade et al., 2004). Chardon et al. (2004) identified 62 MQTLs related to flowering time in maize by synthesizing 313 QTLs from different mapping populations and two important MQTL clusters for flowering time in bins 8.05 and 10.04. Subsequent positional cloning and association mapping analysis showed that *Vgt1*, which is located in bin 8.05, plays an important role in flowering time and the number of nodes (Salvi et al., 2007, 2011). Another MQTL for flowering time, located in bin 10.04, was found along with the key flowering time gene *ZmCCT*, which contains a CCT domain (Hung et al., 2012). These successful examples confirm that meta-analysis is a very useful method for predicting candidate genes and developing molecular markers for complex quantitative traits in maize. In fact, previous studies have identified many MQTLs for yield-related traits by integrating different QTL datasets from maize and many maize orthologs of rice yield-related genes using bioinformatic techniques (Semagn et al., 2013; Wang et al., 2013, 2016; Martinez et al., 2016). However, the number of QTLs that have been integrated in previous studies is still low, and methods for effectively mining candidate loci or genes for yield-related traits from these MQTL regions are still not well-developed.

A combination of linkage mapping and association mapping has recently proven to be an efficient method for identifying candidate loci related to yield-related traits in maize. Multiple major QTLs related to kernel size and weight, such as *qKS2, qGW4.05, qKL1.07*, and *qKW7.02*, were identified through linkage mapping, and their locations were subsequently narrowed down to very small genomic regions through association mapping (Chen et al., 2016a; Li et al., 2016b; Qin et al., 2016; Zhang et al., 2017). Similar to this strategy, combining QTL meta-analysis and regional association mapping to mine MQTLs and narrow down their associated confidence intervals has been suggested as a quick and effective way to identify candidate functional genes or loci (Daware et al., 2017). Based on this strategy, two potential candidate genes for grain size and weight were successfully identified in rice (Daware et al., 2017). Here, we use this strategy in maize to achieve the following objectives: (1) to synthesize the information on QTLs for grain yield and related traits published between 2000 and 2016; (2) to mine MQTLs across the entire genome through QTL meta-analysis; (3) to identify maize orthologs of rice yield-related genes using a comparative genomics strategy; and (4) to identify candidate genes or loci for kernel-related traits by combining meta-analysis with regional association mapping.

## Materials and methods

### QTL data collection for maize yield and related traits

QTL data on maize grain yield (GY) and GY-related traits were collected from 33 studies published between 2000 and 2016 (Table S1). The GY-related traits included two important components: (1) ERT, including ear weight (EW), ear length (EL), ear diameter (ED), cob weight (CW), cob diameter (CD), and kernel row number (KRN); and (2) kernel-related traits (KRT), including kernel length (KL), kernel width (KWI), kernel thickness (KT), kernel number (KN), kernel weight (KW), kernel ratio (KR), and kernel volume (KV) (Table 1). Detailed QTL information and the associated literature on maize grain yield and related traits were collected from three databases, MaizeGDB (http://www.maizegdb.org), Gramene (http://www.gramene.org), and the PubMed web server (http://www.ncbi.nlm.nih.gov/pubmed). In this study, we collected only QTLs that were identified under normal growth conditions.

**Table 1:** List of traits evaluated in this study.

	**Trait name**	**Acronym**	**Traits included^a^**	**No. of populations**	**No. of QTLs**
Yield	Grain Yield	GY	Grain yield per plant Grain yield per plot	25	142
**EAR-RELATED TRAITS**					
	Cob Weight	CW	Cob weight Cob dry weight	3	17
	Ear Weight	EW	Ear weight Ear dry weight	7	33
	Kernel Row Number	KRN	Ear row number Kernel row number	11	92
	Cob Diameter	CD	Cob diameter	2	15
	Ear Length	EL	Ear length	11	68
	Ear Diameter	ED	Ear diameter	9	52
**KERNEL-RELATED TRAITS**					
	Kernel Length	KL	Kernel length 10-Kernel length 20-Kernel length	7	62
	Kernel Width	KWI	Kernel width 10-Kernel width 20-Kernel width	7	76
	Kernel Thickness	KT	Kernel thickness 10-Kernel thickness 20-Kernel thickness	7	76
	Kernel Number	KN	Kernel number per plant Kernel number per row Kernel number per ear	20	109
	Kernel Weight	KW	Hundred kernel weight 300-Kernel weight Thousand kernel weight	28	218
	Kernel Ratio	KR	Kernel ratio	3	11
	Kernel Volume	KV	Kernel volume	4	28

a *Traits identified from the surveyed papers that were included in the same category in our study*.

### Meta-analysis

The collected QTLs were projected onto the “IBM2 2008 Neighbors” maize reference map (http://curation.maizegdb.org/cgi-bin/displaymaprecord.cgi?id=1140201) to generate a consensus map. Then, a meta-analysis was performed to integrate the QTL data from different studies and to refine the confidence intervals. For the meta-analysis of many QTLs, five different models (1-, 2-, 3-, 4-, or N-QTL) with different Akaike information criterion (AIC) values have been proposed using BioMercator software V4 (http://moulon.inra.fr/index.php/en), where the model with the lowest AIC-value is considered optimal. Finally, the consensus QTL presented by the optimum model is regarded as the MQTL (Arcade et al., 2004).

### Identification of annotated transcripts and gene ontology analysis

The physical intervals of these MQTLs were identified using the MaizeGDB and Gramene databases. The physical locations of flanking markers for these MQTLs were confirmed on the IBM2 2008 Neighbors map. The annotated transcripts within these MQTL regions were mined in the MaizeGDB database. The physical locations of these MQTL regions were based on genome annotation version AGPv2 of the maize B73 reference map (http://curation.maizegdb.org/). The gene sequences located in the MQTL regions were aligned to the NCBI non-redundant (nr) database with Blastx, using an *E*-value of < 10^−5^, with a hit number threshold of 100. The best functional annotations were obtained using this process. With the Nr annotation, we used the Blast2GO program (version: v2.5.0) to obtain the gene ontology (GO) annotation for these genes.

### Mining of homologous genes in the MQTL regions

In this study, we collected 25 genes related to grain yield and related traits in rice (Table S3). Homologous genes in maize were identified as follows: (1) the protein sequences of these 25 collected rice genes were obtained from http://www.ricedata.cn/gene; (2) BLASTP (protein-protein BLAST) searches using these protein sequences were performed against the maize “non-redundant protein sequences” database (https://www.ncbi.nlm.nih.gov/). The criteria for these searches included an *E*-value of < 10^−10^, identity >60%, and a coverage region >60% to select homologous genes of these rice yield-related genes in maize.

### Regional association mapping and expression analysis of the candidate genes

An association mapping panel with 627 maize inbred lines covering highly diverse maize germplasms was applied in this study to perform regional association mapping. The genotypes and phenotypes of this association panel have been provided in our previous reports (Chen et al., 2016a; Qin et al., 2016). We selected SNP markers with minor allele frequencies >0.05 in MQTL regions to perform the analysis. The association analysis was estimated using a mixed linear model (MLM) incorporated in TASSEL V5.0, controlling for population structure (Q) and kinship (K). The first three principal components (PCs), which have been analyzed in previous studies, were used as the covariant variables to control for the existing population structure in the association mapping panel. Significant marker-trait associations were declared at LOD >3. The expression data from different kernel development values of these candidate genes were collected from the MaizeGDB database (http://maizegdb.org/) (Winter et al., 2007; Sekhon et al., 2011).

## Results

### QTL collection for maize grain yield and its related traits

**Figure 1:**
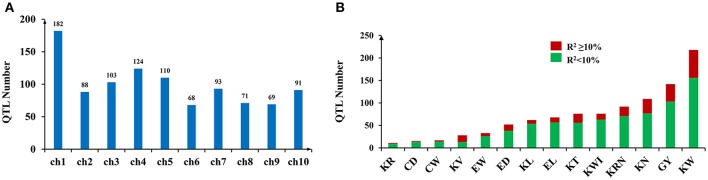
QTL numbers and associated *R*^2^-values (variance explained by a single QTL) distributed across the entire genome. **(A)** QTL numbers distributed on each chromosome. **(B)** The variance explained by a single QTL in the traits included in this study.

A total of 999 QTLs related to GY, ERT, and KRT were collected (Table S1). The number of QTLs per trait ranged from 11 (kernel ratio, KR) to 218 (kernel weight, KW) (Table 1). The 999 collected QTLs were distributed unevenly across the ten chromosomes (Figure 1A). The greatest number of QTLs (182) were located on chromosome 1, while chromosome 6 exhibited the fewest, with 68 (Figure 1A), similar to previous meta-analyses of maize yield QTLs (Wang et al., 2016). A total of 75.08% of the collected QTLs for each trait exhibited an *R*^2^ < 10%, implying that the proportion of phenotypic variance explained by each QTL was very small (Figure 1B). These results suggest that grain yield and related traits in maize are mainly controlled by numerous loci of minor effect and display a complex genetic architecture.

### QTL meta-analysis

**Figure 2:**
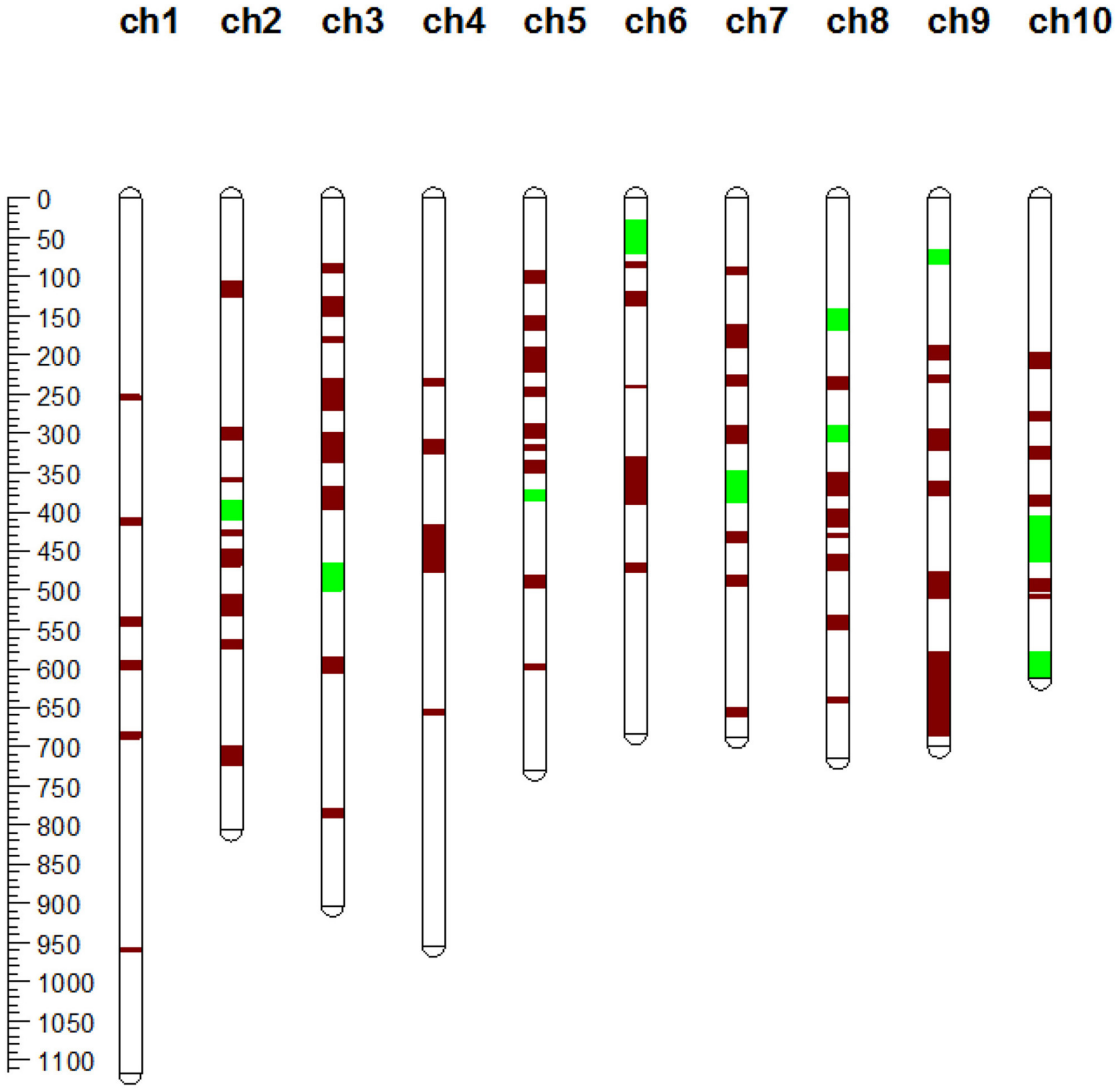
Chromosomal locations of MQTLs identified in this study. The red segments on the chromosome represent MQTLs related to grain yield (GY), ear-related traits (ERT), and kernel-related traits (KRT), while the green segments represent MQTLs related to KRT alone or to GY and KRT simultaneously.

The collected QTLs for maize yield and its related traits were projected onto the target map IBM2 2008 Neighbors via meta-analysis to mine MQTLs and refine QTL intervals. A total of 76 MQTLs were identified according to the models with the lowest AIC-values (Table 2, Figure 2). These MQTLs were distributed unevenly across the genome, with the number per chromosome ranging from 4 on chromosome 4–10 on chromosome 5 (Table 2, Figure 2). These MQTLs were named sequentially from MQTL-1 to MQTL-76 according to their chromosomal locations. MQTL-31 covered just two QTLs, while MQTL-28 interestingly covered 45 QTLs (Table 2). We also found that 60 MQTLs were related to GY, while 4 MQTLs were related to only kernel-related traits (KRT), and MQTL-17, on chromosome 3, was associated with only ERTs (Table 2).

**Table 2:** QTL meta-analysis results.

**MetaQTL name**	**Chr**	**MetaQTL position^a^**	**Left marker**	**Right marker**	**MetaQTL interval (cM)**	**Physical distance (Mb)^b^**	**MetaQTL interval (kb)**	**QTLs^c^**	**Traits^d^**
MQTL-1	1	252.82	umc1222	gpm556	250.4–255.24	10.99~12.22	1231.72	45	ERT, GY, KRT
MQTL-2	1	411.32	imd1	ts2	407.16–415.48	44.53~46.68	2150.47	28	ERT, GY, KRT
MQTL-3	1	541.08	dupssr26	IDP1986	536.72–545.44	73.8~83.43	9632.97	18	ERT, GY, KRT
MQTL-4	1	596.16	AY110396	umc1611	590.48–601.84	147.99~152.17	4175.67	13	ERT, GY, KRT
MQTL-5	1	685.51	npi429	bnl34	681.18–689.84	187.98~191.13	3157.88	36	ERT, GY, KRT
MQTL-6	1	959.5	IDP8008	pco106440	957.04–961.96	250.02~254.38	4363.29	42	ERT, GY, KRT
MQTL-7	2	115.66	umc1542	IDP8711	105.94–125.38	4.67~5.52	852.21	12	ERT, KRT
MQTL-8	2	299.88	umc34	hct5	291.96–307.8	28.21~31.83	3619.91	19	ERT, GY, KRT
MQTL-9	2	359.01	IDP1415	umc1861	355.95–362.07	48.42~52.01	3588.19	10	ERT, GY, KRT
MQTL-10	2	397.49	IDP496	pza03211	385.26–409.72	62.92~149.11	86187.65	8	KRT
MQTL-11	2	427.36	bnlg1396	emp2	424.56–430.16	154.53~177.65	23125.51	15	ERT, GY, KRT
MQTL-12	2	458.67	umc1108	pza03529	448.06–469.28	186.53~189.45	2914.67	6	ERT, KRT
MQTL-13	2	518.88	IDP3824	umc1745	506.08–531.68	199.17~205.84	6668.02	5	ERT, KRT
MQTL-14	2	568.83	AI668346	IDP136	563.93–573.73	209.83~211.52	1683.62	8	ERT, KRT
MQTL-15	2	711.39	bnlg469b	IDP7539	699.76–723.02	231.21~233.26	2050.58	5	ERT, GY, KRT
MQTL-16	3	89.42	IDP2399	umc1458	84.74–94.1	3.84~4.68	840.57	15	ERT, KRT
MQTL-17	3	137.31	IDP5966	lim66	125.74–148.88	7.67~10.08	2405.79	6	ERT
MQTL-18	3	179.79	ra2	gpm697	176.06–183.52	12.88~15.04	2159.08	14	ERT, GY, KRT
MQTL-19	3	249.5	bnlg2047	IDP7433	230.84–268.16	31.06~63.84	32779.45	14	ERT, KRT
MQTL-20	3	317.83	umc1750	cdo250	299.86–335.8	86.75~136.09	49332.30	10	ERT, GY
MQTL-21	3	382.41	vp1	pza00667	368.13–396.69	162.74~162.8	63.89	9	ERT, GY, KRT
MQTL-22	3	482.81	bnlg1047a	umc1644	465.23–500.39	178.14~183.89	5743.96	5	KRT, GY
MQTL-23	3	596.27	cl23834_1	AY106518	586.8–605.74	201.17~205.26	4088.86	17	ERT, GY, KRT
MQTL-24	3	785.31	IDP7267	IDP6978	780.33–790.29	221.62~222.57	950.52	13	ERT, GY, KRT
MQTL-25	4	234.99	umc1758	phm3301	231.91–238.07	4.75~5.33	580.77	25	ERT, GY, KRT
MQTL-26	4	316.25	umc2281	bnl5.46a	307.59–324.91	17.24~17.85	615.25	21	ERT, GY, KRT
MQTL-27	4	446.34	gpm155	AY110355	416.01–476.67	39.32~144.04	104725.68	33	ERT, GY, KRT
MQTL-28	4	656.15	pzb01461	IDP4308	653.89–658.41	186.37~188.2	1827.71	45	ERT, GY, KRT
MQTL-29	5	99.91	TIDP3193	sqs1	93.17–106.65	2.8~3.68	882.55	11	ERT, GY, KRT
MQTL-30	5	159.31	gpm160	pza02753	151.14–167.48	6~7.67	1674.02	12	ERT, GY, KRT
MQTL-31	5	205.67	umc1587	ago108	189.6–221.74	10.17~13.61	3432.94	2	ERT, KRT
MQTL-32	5	246.46	smh6	bnl7.56	241.89–251.03	17.1~20.92	3820.54	6	ERT, KRT
MQTL-33	5	297.2	umc2295	IDP7018	288.85–305.55	38.17~61.53	23357.32	15	ERT, GY, KRT
MQTL-34	5	317.51	cdpk1	umc1226	314.88–320.14	60.8~69.15	8346.70	3	ERT, KRT
MQTL-35	5	341.37	csu315a	lox11	334–348.74	78.36~123.21	44843.81	20	ERT, GY, KRT
MQTL-36	5	378.91	TIDP3443	amp3	372.76–385.06	162.84~167.47	4632.00	11	GY, KRT
MQTL-37	5	488.62	TIDP8870	IDP758	481.3–495.94	188.65~193.45	4795.04	15	ERT, GY, KRT
MQTL-38	5	597.13	umc68a	gpm874b	594.19–600.07	205.44~207.73	2289.51	15	ERT, GY, KRT
MQTL-39	6	48.85	umc2310	gpm399a	27.67–70.03	0.5~21.9	21403.88	14	GY, KRT
MQTL-40	6	85.03	TIDP3648	nfa101	82.25–87.81	17.96~28.25	10292.78	9	ERT, GY, KRT
MQTL-41	6	127.25	mmp108b	php20045a	119.25–135.25	36.56~89.15	52587.33	14	ERT, GY, KRT
MQTL-42	6	240.02	TIDP3136	cl5367_1b	238.19–241.85	118.79~120.66	1874.75	13	ERT, KRT
MQTL-43	6	359.68	chr116a	umc1859	329.64–389.72	146.06~154.5	8434.75	6	ERT, GY, KRT
MQTL-44	6	471.13	dupssr15	asg47	466.31–475.95	162.24~163.11	870.04	12	ERT, GY, KRT
MQTL-45	7	93.44	psk1	bnlg2132	89.69–97.19	3.02~3.25	223.13	7	ERT, GY, KRT
MQTL-46	7	175.66	pco086679	in1	161.5–189.82	9.78~19.36	9577.94	4	ERT, KRT
MQTL-47	7	233.31	psr371b	TIDP2647	226.64–239.98	19.44~34.19	14743.05	18	ERT, GY, KRT
MQTL-48	7	301.69	umc1138	IDP4794	291.09–312.29	105.91~122.71	16803.73	20	ERT, GY, KRT
MQTL-49	7	367.49	TIDP2699	mmp152	347.16–387.82	131.79~140.69	8900.18	20	KRT
MQTL-50	7	431.93	psr371a	rz596a	425.01–438.85	147.02~154.31	7295.23	4	ERT, GY, KRT
MQTL-51	7	486.95	IDP6922	IDP5024	480.55–493.35	160.14~161.84	1699.20	15	ERT, GY, KRT
MQTL-52	7	656.1	umc168	kin1	651.64–660.56	170.25~176.22	5970.54	5	ERT, GY, KRT
MQTL-53	8	154.62	mmp85	phm9695	142.15–167.09	8.1~12.29	4189.31	8	GY, KRT
MQTL-54	8	235.1	IDP7228	fps1	227.58–242.62	22.98~63.28	40298.35	15	ERT, GY, KRT
MQTL-55	8	299.61	IDP8347	gpm599b	290.08–309.14	96.17~100.91	4737.17	7	GY, KRT
MQTL-56	8	364.84	AY110056	hox1	351–378.68	112.68~123.91	11235.54	9	ERT, GY, KRT
MQTL-57	8	408.35	phm10525	thi1	397.46–419.24	126.08~138.14	12067.77	10	ERT, GY, KRT
MQTL-58	8	430.67	csu31a	umc2210	429.13–432.21	146.89~160.45	13555.71	7	ERT, GY, KRT
MQTL-59	8	465.26	umc1149	ald2	455.7–474.82	160.3~163.31	3012.28	7	ERT, GY, KRT
MQTL-60	8	541.64	csu110c	npi414a	532.71–550.57	168.25~169.79	1538.48	5	ERT, GY, KRT
MQTL-61	8	639.57	AY110127	phi233376	637.02–642.12	173.41~175.44	2034.87	3	ERT, GY
MQTL-62	9	74.32	bnlg1724	php10005a	66.04–82.6	4.3~5.73	1427.18	9	GY, KRT
MQTL-63	9	196.6	klp1c	pza00860	188.89–204.31	16.24~18.61	2365.98	7	ERT, GY, KRT
MQTL-64	9	229.76	umc1698	eps1	225.67–233.85	19.2~22.68	3481.87	7	ERT, KRT
MQTL-65	9	307.2	magi67004	gpm165	294.85–319.55	90.84~107.89	17045.02	11	ERT, GY, KRT
MQTL-66	9	371.1	umc1492	umc1387	361.61–380.59	120.2~133.6	13393.58	16	ERT, GY, KRT
MQTL-67	9	493.46	IDP3889	mmp131	477.47–509.45	140.18~144.79	4605.29	14	ERT, GY, KRT
MQTL-68	9	631.76	IDP2142	rld1	579.19–684.33	147.35~154.65	7298.10	5	ERT, GY, KRT
MQTL-69	10	206.79	npi105a	umc1962	197.75–215.83	13.06~24.61	11558.08	12	ERT, GY, KRT
MQTL-70	10	277.58	bnlg640	umc1246	272.86–282.3	85.27~102.52	17256.90	15	ERT, GY, KRT
MQTL-71	10	324.42	lox7	magi13270	316.63–332.21	120.22~126.49	6277.80	12	ERT, GY, KRT
MQTL-72	10	385.24	IDP6861	gpm522b	378.31–392.17	133.55~136.1	2550.91	17	ERT, GY, KRT
MQTL-73	10	434.86	TIDP4639	IDP4016	405.79–463.93	136.94~143.07	6125.36	4	KRT
MQTL-74	10	492.99	AY110016	IDP167	485.02–500.96	144.47~148.54	4067.76	12	ERT, GY, KRT
MQTL-75	10	508.33	IDP2352	csu300b	506.4–510.26	148.88~149.07	192.64	13	ERT, GY, KRT
MQTL-76	10	594.48	gpm23b	umc1645	578.71-610.25	147.97~149.79	1821.60	6	KRT

a *The most likely position of the MQTL in the IBM2 2008 Neighbors map*.

b *The physical confidence intervals of the MQTLs are based on B73 ref V2 and the corresponding position on B73 ref V4 are listed in Table S5*.

c *The number of QTLs contained in the MQTL regions*.

d *GY, grain yield; ERT, ear-related traits; and KRT, kernel-related traits. If an MQTL contained only a QTL for kernel-related traits, we referred to it as a KRT MQTL*.

Furthermore, we found that several yield-related MQTLs tended to show a clustered distribution in the maize genome. One of these clustered regions was detected in the genomic region from 38.17 to 69.15 Mb on maize chromosome 5 and consisted of two MQTLs (MQTL-33 and MQTL-34) (Table 2). Two MQTLs (MQTL-39 to MQTL-40) were clustered in a 28.5-Mb region on maize chromosome 6 (Table 2). Other clusters were found on maize chromosome 7 (from 9.78 to 34.19 Mb), chromosome 8 (from 146.89 to 163.31 Mb), and chromosome 9 (from 16.24 to 22.68 Mb) (Table 2). Five MQTLs (from MQTL-72 to MQTL-76) were clustered in a 16.24-Mb genomic region on maize chromosome 10 (Table 2).

### Identification of annotated transcripts and go analysis in MQTL regions

**Figure 3:**
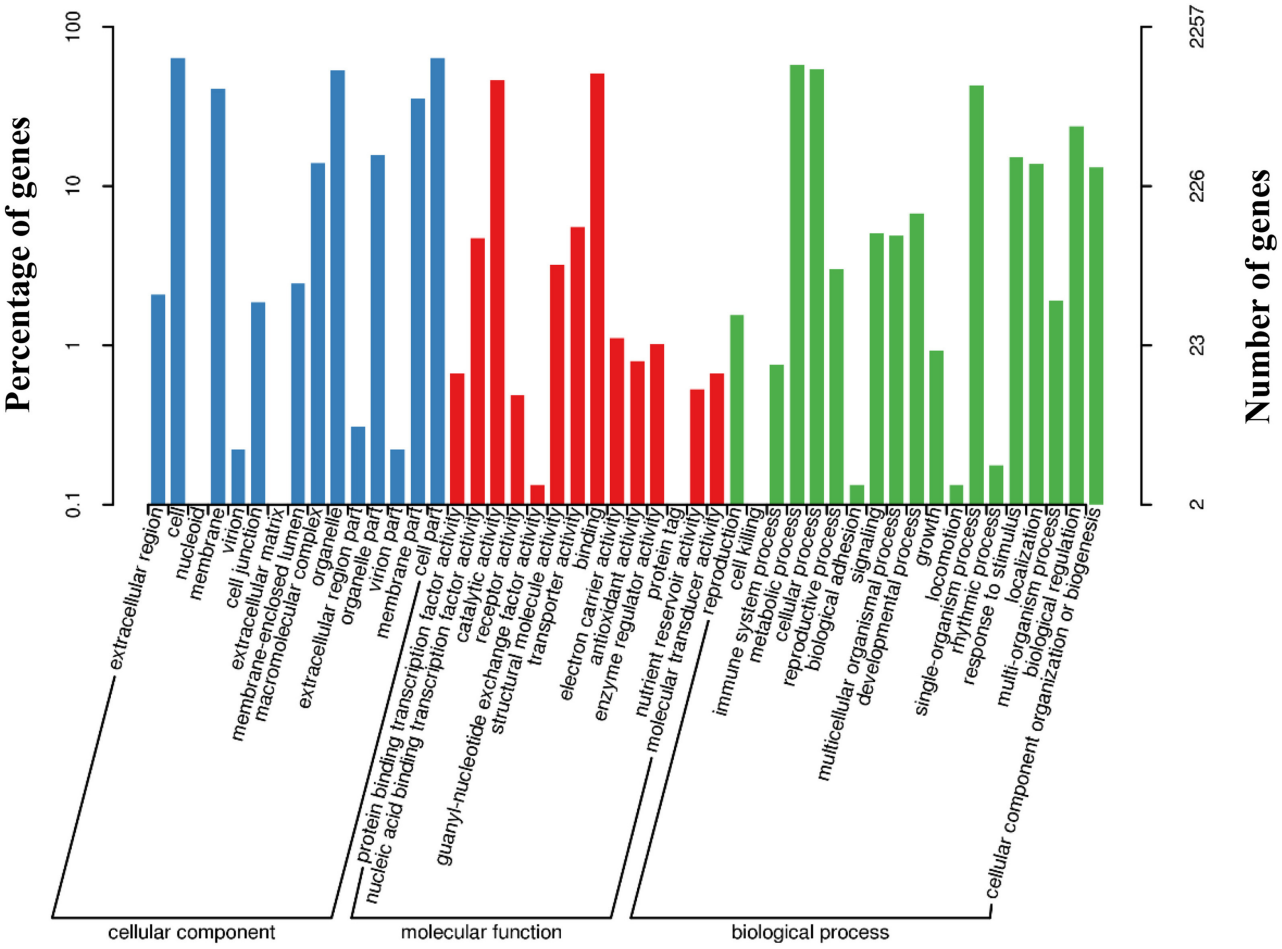
Gene ontology analysis of the genes located within a 5 Mb interval of the MQTL regions.

Among the 76 MQTLs, 44 MQTLs with an interval of less than 5 Mb were selected for GO analysis of annotated transcripts. A total of 4,501 genes were identified in the 44 MQTL regions, whose detailed gene IDs are presented in Table S2. Multiple genes associated with yield-related traits in maize were found to be located in MQTL intervals. *Fea2* (Bommert et al., 2013) and *Ub2* (Chuck et al., 2014), which are important genes in the control of kernel row numbers in maize, were located in the MQTL-27 and MQTL-5 regions, respectively. The *UBL1* gene, which plays an important role in kernel and seedling development by influencing pre-mRNA splicing (Li et al., 2016a), was located in MQTL-37. Three genes related to maize kernel development, *emp2* (Fu et al., 2002), *ZmZHOUPI* (Grimault et al., 2015), and *ZmReas1* (Qi et al., 2016), were located in the MQTL-11, MQTL-9, and MQTL-43 regions, respectively. According to the two-level WEGO classification, 2,257 genes were annotated, which were divided into three categories (cellular component, molecular function, and biological pathway) and further divided into 48 classes (Figure 3). We found that more than one thousand genes were related to the cell, cell part, metabolic process, cellular process, organelle, binding, and catalytic activity terms (Figure 3).

### Homologous gene mining in MQTL regions

To identify candidate genes related to grain yield in the MQTL regions, we collected 25 genes that were functionally characterized as being associated with grain yield and its components from the rice genome (Table S3). Homologous genes were not found in the maize genome for four of these rice genes (*APG, GW5, PGL1, qGL3*), while eight of the rice genes (*An-1, Bsg1, D2, DEP1, GIF1, GW2, LP, SMG1, SRS3*, and *SRS5*) exhibited more than one homologous gene, and the other genes exhibited a single homologous gene in maize (Table S3). Finally, 11 MQTL regions containing 11 maize orthologs of 10 rice genes related to grain yield and its related traits were identified in this study.

MQTL-19 contained *ZmGS5* (Gene ID: GRMZM2G13815), an ortholog of the well-characterized rice yield gene *GS5* (Table 3). In rice, *GS5* encodes a serine carboxypeptidase family protein that controls grain size and weight by regulating grain width and filling (Li et al., 2011). One candidate gene (Gene ID: GRMZM2G097275), homologous to *OsSPL14*, was identified in the MQTL-35 region (Table 3). *OsSPL14* is a member of the SBP (squamosa promoter-binding-like transcription activator) family, and can improve the grain yield by regulating plant architecture (Miura et al., 2010). *GN1a* encodes a cytokinin dehydrogenase 2 protein and regulates grain number to improve grain yield in rice (Ashikari et al., 2005). A *GN1a* homolog (Gene ID: GRMZM2G325612) was identified in maize in the MQTL-54 region (Table 3). A homologous gene of *D61* (Gene ID: GRMZM2G048294), which encodes a brassinosteroid receptor kinase and can regulate plant height to produce small grains in rice (Morinaka et al., 2006), was identified in the MQTL-58 region (Table 3). Homologs of other grain yield-related genes, such as *LP1, SRS5, Ghd7*, and *DEP1*, were also identified in MQTL regions (Table 3). These homologs of yield-related genes may play important roles in ear and kernel development in maize.

**Table 3:** Candidate genes associated with yield-related traits in metaQTL regions.

**Gene name**	**Accession number**	**Gene product**	**Trait in rice**	**Homologous gene ID in maize**	**Corresponding metaQTL region**	**References**
*Bsg1*	LOC_Os02g56610	DUF640 domain containing protein	Grain size and grain weight	GRMZM2G147241	MQTL-8	Ren et al., 2016
*An-1*	LOC_Os04g28280	Basic helix-loop-helix protein	Awn development, grain size, and grain number	GRMZM2G137541	MQTL-12	Luo et al., 2013
*GS5*	LOC_Os05g06660	Peptidase S10, serine carboxypeptidase family protein	Grain size and grain weight	GRMZM2G13815	MQTL-19	Li et al., 2011
*OsSPL14*	LOC_Os08g39890	Squamosa promoter-binding-like transcription activator	Grain yield, plant architecture	GRMZM2G097275	MQTL-35	Miura et al., 2010
*DEP1*	LOC_Os09g26999	Phosphatidylethanolamine-binding protein (PEBP) like domain protein	Grain yield, panicle length, and grain size	GRMZM2G172320	MQTL-48	Sun et al., 2014
*SRS5*	LOC_Os11g14220	Alpha-tubulin protein	Grain size	GRMZM2G083243	MQTL-51	Segami et al., 2012
				GRMZM2G051782	MQTL-67	
*GN1a*	LOC_Os01g10110	Cytokinin dehydrogenase 2	Grain number and grain yield	GRMZM2G325612	MQTL-54	Ashikari et al., 2005
*D61*	LOC_Os01g52050	Brassinosteroid LRR receptor kinase	Grain yield and plant height	GRMZM2G048294	MQTL-58	Morinaka et al., 2006
*LP1*	LOC_Os09g28300	Remorin, C-terminal region domain containing protein	Panicle length	GRMZM2G442489	MQTL-65	Liu et al., 2016
*Ghd7*	LOC_Os07g15770	CCT domain protein	Grain number, plant height and flowering time	GRMZM2G381691	MQTL-70	Xue et al., 2008

### Regional association analysis underlying KRT MQTL regions

In our study, a total of 10 MQTL regions were found to be related to KRT alone or to both GY and KRT (Table 2). These 10 MQTL regions were selected to implement a regional association analysis. The integration of SNP genotyping data from the 10 selected MQTL regions with phenotype information (10-kernel length, 10KL; 10-kernel width, 10KW; 100-kernel weight, HKW) for all 627 accessions successfully revealed significant associations for four MQTL regions (MQTL-10, MQTL-39, MQTL-49, and MQTL-73).

#### MQTL-10 region

**Figure 4:**
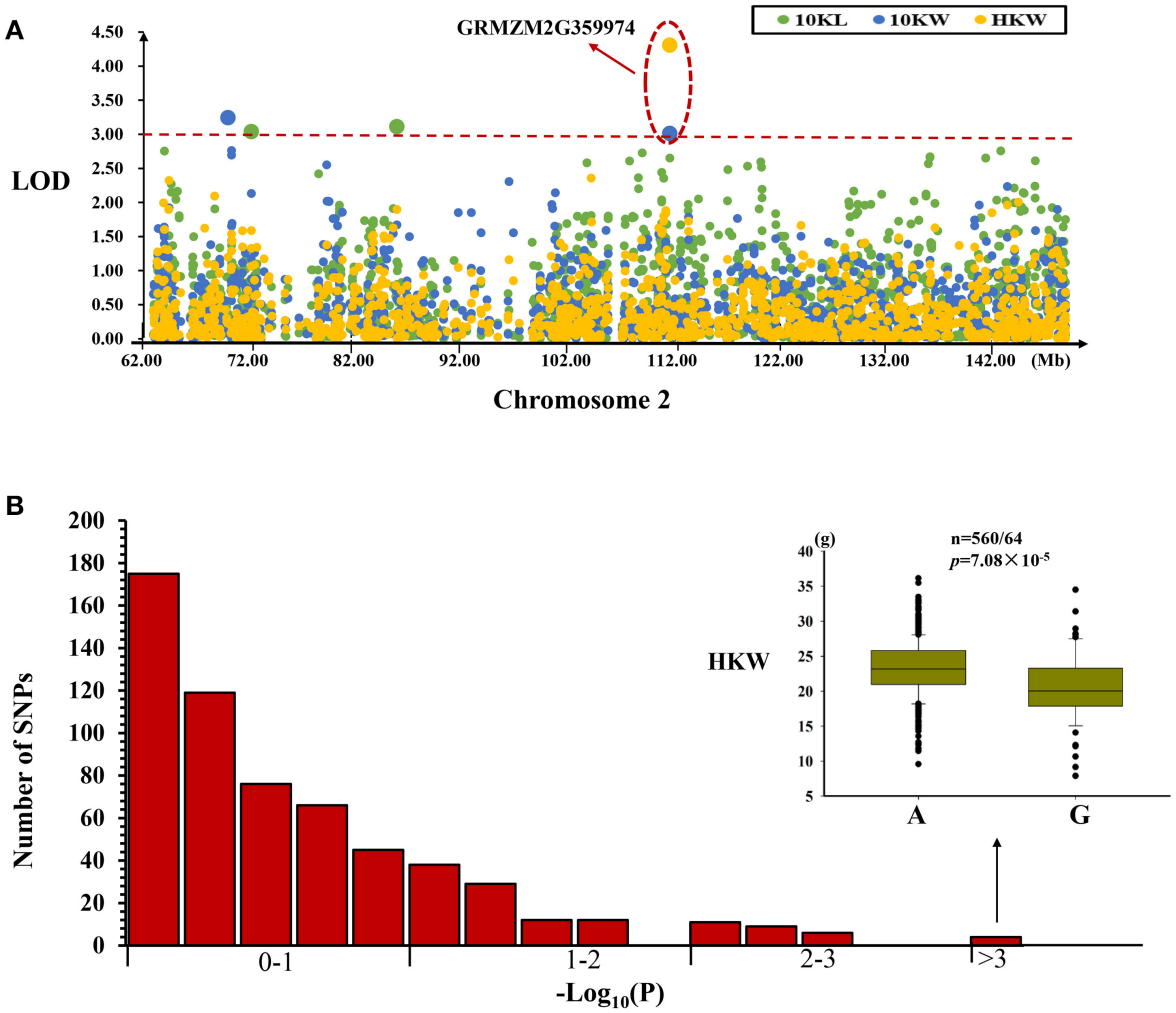
Regional association analysis of the MQTL-10 region. **(A)** Association analysis between the SNPs located within the MQTL-10 region and kernel size and weight. The green, blue, and yellow circles represent different SNPs, whereas the red line represents the significance threshold. An LOD >3 indicates that a SNP is significantly related to kernel size or weight. One SNP located in the gene (GRMZM2G359974) was found to be associated with kernel width and weight. **(B)** Permutation test for the identified significantly associated SNP markers. Comparison of the most highly significant SNP (*n* = 560/64, *P* = 7.08 × 10^−5^) with the association results for 600 randomly selected SNPs for the same trait. These results suggested that the significant association was a real association and was not caused by a false positive.

We selected SNP markers in an interval (chr2: 62924210~149111857) corresponding to the MQTL-10 region in the maize genome. Using a mixed linear model, we identified two, two and one SNPs associated with variation in kernel length, kernel width, and hundred kernel weight, respectively (Table 4, Figure 4A). These significant SNP loci explained 2.72–3.80% of the observed phenotypic variation in kernel size and weight in this association mapping panel (Table 4). We also identified one SNP (PZE-102096886) located in the GRMZM2G359974 gene that was associated with the variation in kernel weight and 10-kernel width (Figure 4A). Comparisons of these significant *P*-values with the *P*-value distribution of 600 randomly chosen SNPs from this region suggested that these significant associations were not due to false positives (Figure 4B).

**Table 4:** Summary of SNP-based regional association analysis in metaQTL regions.

**MQTL name**	**Trait^a^**	**SNP**	**Chr**	**Pos**	**LOD**	**PVE %**	**Allele**	**Structural annotation**
MQTL-10	HKW	PZE-102096886	2	111583492	4.31	3.80	A/G	GRMZM2G359974
	10KL	PZE-103095280	2	85861839	3.11	2.81	A/G	Intergenic
	10KL	PZE-102084262	2	72146069	3.04	2.74	A/C	Intergenic
	10KW	PZE-102083125	2	69945211	3.24	2.92	A/G	Intergenic
	10KW	PZE-102096886	2	111583492	3.01	2.72	A/G	GRMZM2G359974
MQTL-39	HKW	SYN11458	6	9494055	3.10	2.80	A/G	GRMZM2G301884
	10KW	PZE-106021411	6	18990406	3.01	1.81	A/G	Intergenic
MQTL-49	10KW	SYN35079	7	132632787	3.17	2.85	A/G	GRMZM2G083894
MQTL-73	HKW	PZE-110091041	10	140189920	3.35	3.01	A/T	Intergenic

a *HKW, Hundred kernel weight; 10KL, 10-kernel length; 10-KW, 10-kernel width*.

#### MQTL-39 region

**Figure 5:**
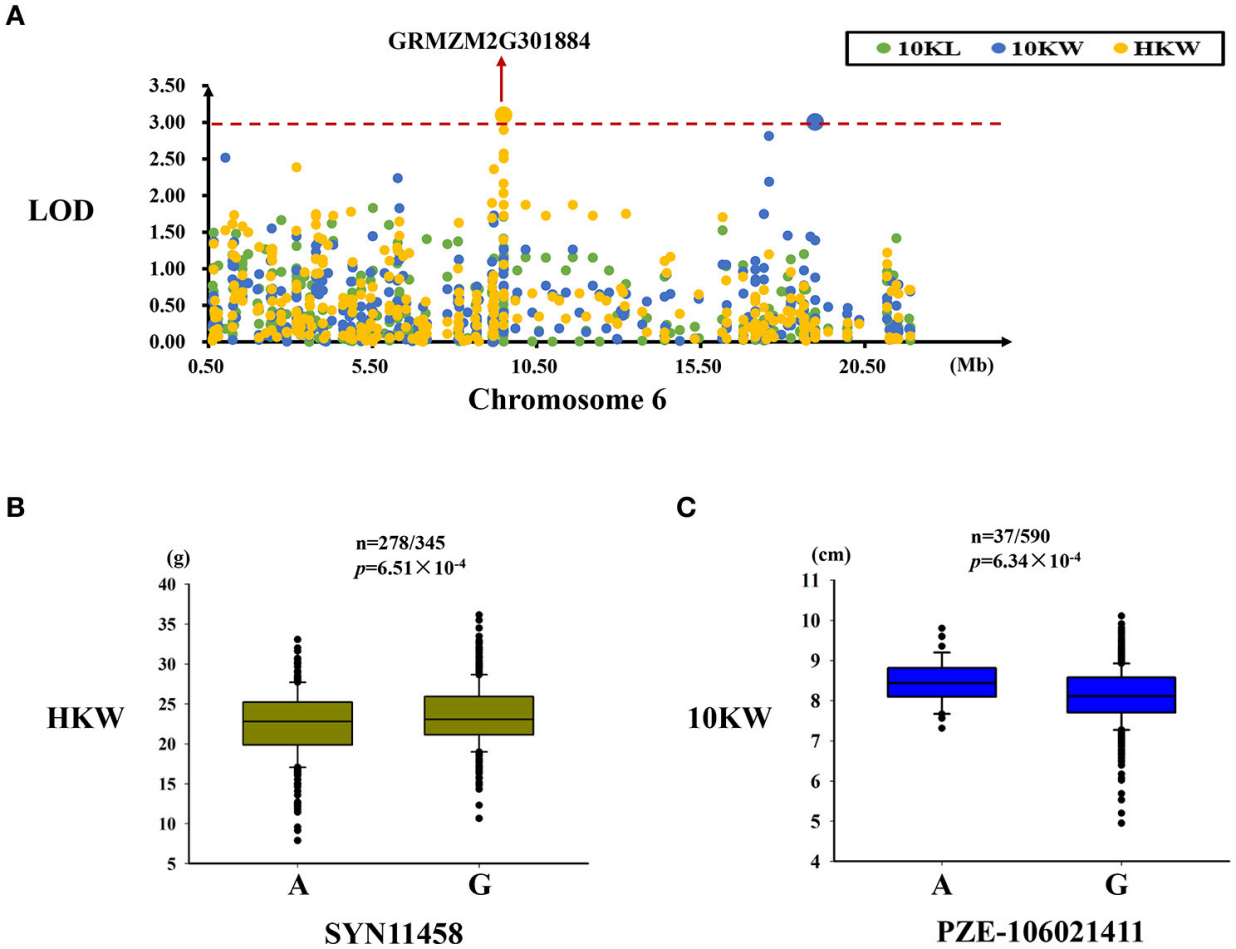
Regional association analysis of the MQTL-39 region. **(A)** Association analysis between the SNPs located in the MQTL-39 region and kernel size and weight. A SNP (SYN11458) associated with kernel weight was found in this gene (GRMZM2G301884), and another SNP (PZE-106021411) associated with kernel width was identified in the intergenic region. **(B)** The SYN11458 SNP was significantly related to kernel weight (*n* = 278/345, *P* = 6.51 × 10^−4^). SYN11458 has two alleles (A and G), and the G allele is associated with a greater kernel weight. **(C)** The SNP PZE-106021411 was significantly related to kernel width (*n* = 37/590, *P* = 6.34 × 10^−4^). PZE-106021411 had two alleles (A and G), and the A allele was associated with a wider kernel. The red dashed line represent the significance threshold (LOD = 3).

Two significant SNPs associated with HKW and 10KW were identified in this region, while explained 2.80 and 1.81% of the observed phenotypic variation, respectively (Table 4). A significant SNP (SYN11458) related to kernel weight variation was located in the GRMZM2G301884 gene (Figure 5A). For SYN11458, two alleles (A/G) were present in the association panel, with the G allele being associated with a higher kernel weight (Figure 5B). The PZE-106021411 SNP, located in an intergenic region, was significantly associated with kernel width. Two alleles for this SNP (A/G) were present in this panel, with the A allele being associated with greater kernel width (Table 4, Figure 5C).

#### MQTL-49 region

**Figure 6:**
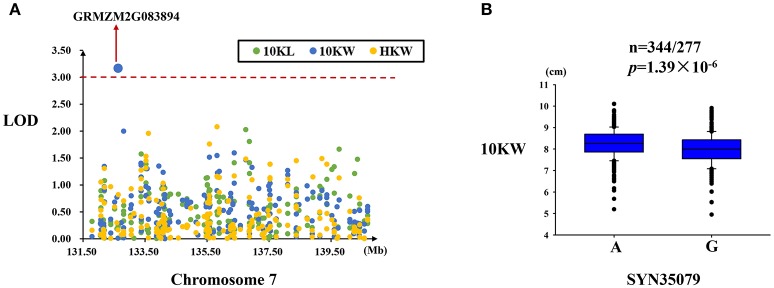
Regional association analysis of the MQTL-49 region. **(A)** Association analysis between the SNPs located in the MQTL-49 region and kernel size and weight. Only one SNP (SYN35079) was found to be associated with kernel width in this gene (GRMZM2G083894). **(B)** The SNP SYN35079 was significantly related to kernel width (*n* = 344/277, *P* = 1.39 × 10^−6^). SYN35079 had two alleles (A and G), and the A allele was associated with a wider kernel. The red dashed line represent the significance threshold (LOD = 3).

Only one significant SNP (SYN35079) located in the GRMZM2G083894 gene was found to be associated with kernel width in this region (Figure 6A). This SNP explained 2.85% of the variation in kernel width and exhibited two alleles (A/G) in this association panel (Table 4). Significant differences in kernel width were found between these two alleles of SYN35079 (Figure 6B).

#### MQTL-73 region

**Figure 7:**
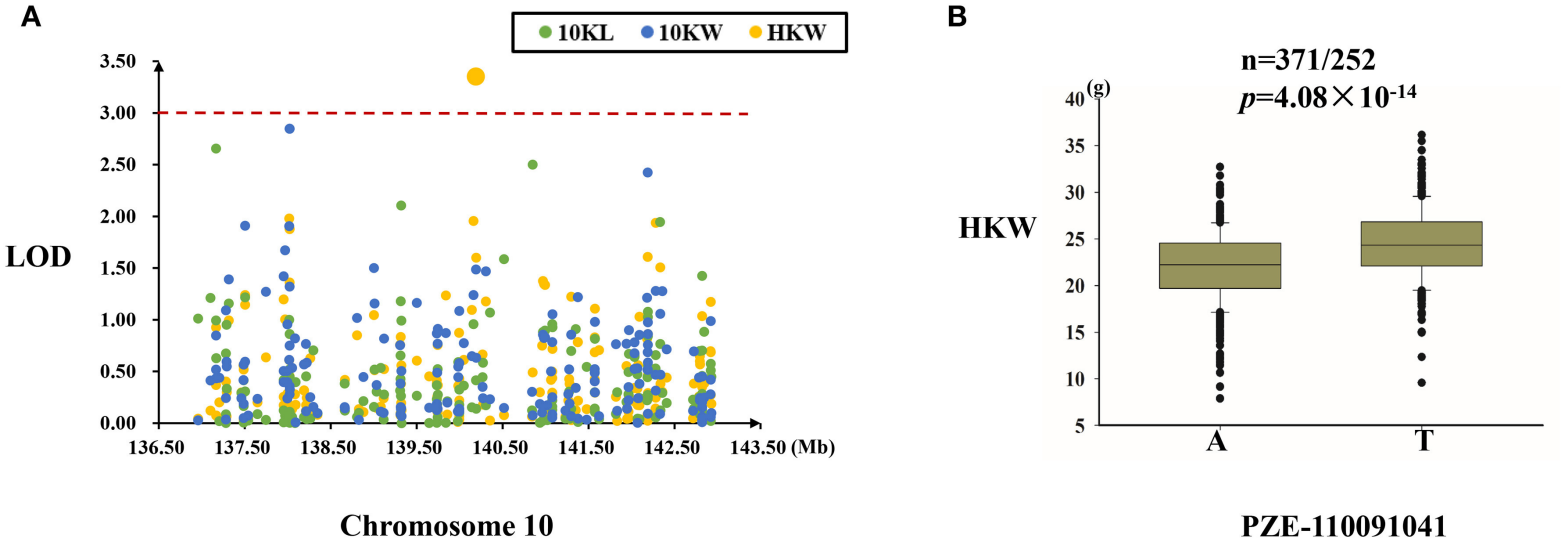
Regional association analysis of the MQTL-73 region. **(A)** Association analysis between the SNPs located in the MQTL-73 region and kernel size and weight. Only one SNP (PZE-110091041) was found to be associated with kernel weight in the intergenic region. **(B)** The PZE-110091041 SNP was significantly related to kernel weight (*n* = 371/252, *P* = 4.08 × 10^−14^). PZE-110091041 had two alleles (A and T), and the T allele was associated with a greater kernel weight. The red dashed line represent the significance threshold (LOD = 3).

One SNP (PZE-110091041), located in the intergenic region, was significantly associated with hundred kernel weight (Figure 7A). This SNP explained 3.01% of the variation in kernel weight, and the T allele of this SNP was associated with a higher kernel weight (Table 4, Figure 7B).

And, we found that the inbred lines which harbor the positive allele in the linkage population also have the SNP allele can increase the corresponding trait (Table S6). *HBqkwid2* is a QTL related with kernel width and found by using the RIL population from the cross of Huangzaosi and Huobai and a positive effect from Huangzaosi (Li et al., 2013). Using regional association mapping, we identified an SNP (A/G) significantly related with kernel width, and Huangzaosi has an “A” allele, which can increase kernel width. The MQTL-73 region contains a hundred kernel weight QTL (*Zqkwei10*), which is found in the RIL populations from Huangzaosi and Zheng58, and the positive effect from Zheng58 can increase the hundred kernel weight (Li et al., 2013). A significant SNP (A/T) was identified using regional association mapping in this region; Zheng58 contains the “T” allele and can increase the kernel weight, and Huangzaosi contains the “A” allele and can decrease the kernel weight.

### Expression pattern analysis of candidate genes

The combination of meta-analysis and regional association mapping indicated that three genes (GRMZM2G359974, GRMZM2G301884, and GRMZM2G083894) may play important roles in maize kernel development. The expression data for the three candidate genes were collected from the MaizeGDB database (http://www.maizegdb.org/). We found that these genes were all expressed at different stages of maize kernel development. For instance, GRMZM2G359974, which encodes a transferase family protein, was highly expressed in endosperm-20 DAP, while GRMZM2G301884 was highly expressed at the early stage of grain development, and GRMZM2G083894, which encodes an AN1-like zinc finger protein, was expressed at different levels in different stages of kernel development in maize (Figure S1). Therefore, these genes should be considered the most promising candidates for involvement in maize kernel development for further functional validation.

## Discussion

### Meta-analysis of yield-related trait QTLs in maize

In maize, most important agronomic traits, such as grain yield, ERT, and kernel-related traits, are complex, quantitative and controlled by numerous small effect QTLs. With increasing numbers of identified QTLs, researchers tend to compare the results of QTL analysis from different backgrounds and environments (Peng et al., 2011; Li et al., 2013). However, the results of QTL mapping are often inconsistent due to different population types, population sizes, mapping methods and genetic backgrounds. Therefore, QTLs localized to inconsistent genomic regions should be investigated further to validate these QTLs and verify their precise positions. Integrating QTL data from different studies and using meta-analysis to identify MQTL regions will pave the way for further fine mapping of QTLs and gene cloning (Wang et al., 2013, 2016; Martinez et al., 2016). In this study, 999 QTLs for maize yield and related traits were collected from different populations (Table S1). Through meta-analysis, a total of 76 maize yield MQTLs were identified in this study (Table 2, Figure 2). Compared with previous studies, we identified many MQTL regions that were highly consistent across different studies (Table S4). We also identified nine genomic regions for yield-related traits that had been identified in previous studies (Wang et al., 2013, 2016; Martinez et al., 2016) (Table S4). Intriguingly, we found that a major QTL (*qGW4.05*) for maize kernel size and weight that we recently fine-mapped was located in the MQTL-27 region on maize chromosome 4. On the same chromosome, a QTL for maize grain yield, *qYPP4-1*, was recently identified using a high-density genetic map, which was placed in the MQTL-28 region by our meta-analysis (Chen et al., 2016b). These results suggest that some MQTL regions identified in our study can serve as major QTLs for fine mapping and gene cloning of important yield genes.

### Homologous gene cloning of maize yield and related traits

In crops with large and complex genomes, such as maize and wheat, homology-based cloning methods are useful tools for identifying important genes related to complex traits. In recent years, many crop genome sequences have been released based on the wide application of next-generation sequencing technologies (Liu et al., 2015). These public genome sequences aid in the identification of conserved genomic regions and key genes in different crops.

With the numerous grain yield-related genes identified in rice, many homologous genes related to grain yield have been cloned in maize based on a comparative genomics strategy. In maize, multiple genes related to kernel size and weight have been identified, and their function has been validated through linkage analysis and association mapping analysis (Li et al., 2010a,b; Liu et al., 2015). *ZmGS5*, a maize homolog of the rice grain size and weight gene *GS5* was identified in the MQTL-19 region in this study (Table 3). Previous studies have demonstrated that brassinosteroids such as *brd2, dwf11*, and *d61* play an important role in seed development in rice. One gene homologous to *D61* was identified in MQTL-58 (Table 3). The dwarf *d61* mutant of rice exhibits smaller grains and a lower grain weight due to defects in brassinolide metabolism (Morinaka et al., 2006). The members of the SBP gene family are important transcription factors in plants, involved in plant growth and development. *OsSPL14*, which encodes an SBP transcription factor, controls shoot branching in the vegetative stage, and a higher grain yield can be achieved in rice via over-expression of this gene in the reproductive stage (Miura et al., 2010). One candidate gene (GRMZM2G097275) was identified in the MQTL-35 region, which was homologous to *OsSPL14* (Table 3). The rice gene *GN1a*, which encodes a cytokinin oxidase, can increase grain number and improve grain yield. When the expression of *GN1a* is reduced, the cytokinin accumulates in inflorescence meristems, and the number of reproductive organs is increased (Ashikari et al., 2005). Furthermore, GRMZM2G325612, a homolog of *GN1a*, was mapped to the MQTL-54 region (Table 3). The homologs of the 10 genes in the MQTL regions have been shown to improve grain yield in rice, suggesting their possible roles in maize. As in previous studies, multiple methods of analysis, such as expression pattern analysis, phylogenetic analysis, linkage analysis, candidate gene mapping analysis, and transgenic analysis, should be used to validate the function of homologous genes. These homologous genes in MQTL regions should to be validated in the future using a number of the techniques mentioned above.

### Combining regional association mapping and MQTL results to mine candidate genes for kernel size and weight

Association mapping and linkage mapping are two effective strategies for dissection of the genetic basis of complex quantitative traits in crops. These two methods each have particular advantages, such as a higher relative power and lower false positive rate for linkage mapping and a relatively higher mapping resolution for association analysis (Sneller et al., 2009). In recent years, the combination of linkage and association mapping strategies has been widely used in crops. One strategy is to construct integrated mapping populations such as MAGIC (multiparent advanced generation inter-crosses) and NAM (nested association mapping) populations (Buckler et al., 2009; Meng et al., 2016). Based on these types of populations, many complex quantitative traits have been resolved, such as flowering time, plant height, and disease resistance (Buckler et al., 2009; Peiffer et al., 2014; Ding et al., 2015). Another strategy is to first determine confidence intervals related to target traits based on linkage mapping analysis. Then, genome-wide association mapping or regional association mapping methods can be used to narrow down the confidence intervals of major QTLs and identify candidate genes or loci. Many major QTLs related to kernel size and weight have been finely mapped to candidate genes in maize (Chen et al., 2016a; Li et al., 2016b; Qin et al., 2016) or rapeseed (Li et al., 2014).

Combining meta-analysis and regional association mapping can rapidly identify candidate genes associated with complex agronomic traits, such as grain size and weight, in rice (Daware et al., 2017). In this study, we identified three candidate genes (GRMZM2G359974, GRMZM2G301884, and GRMZM2G083894) for kernel size and weight by combining meta-analysis and regional association mapping (Table 4, Figures 4–6). One gene (GRMZM2G359974) located in MQTL-10 region encoding a transferase family protein was found to be significantly associated with kernel width and weight through regional association analysis (Table 4, Figure 4). GRMZM2G083894, which encodes an AN1-like zinc finger domain protein, was found to be associated with kernel width and exhibited different expression levels in different stages of kernel development (Table 4, Table S4). The three genes (GRMZM2G359974, GRMZM2G301884, and GRMZM2G083894) are identified by combined the meta analysis and regional association mapping. And, we found the three genes were all expressed at different stages of maize kernel development. To date, many GWAS studies have been done for maize yield and its related traits, but these results are very different for many reasons such as the mapping population, the phenotype variation and the population structure. The three genes identified in our study were near by the significant SNPs with GWAS for kernel size and weight traits through combining 10 RIL populations (Liu et al., 2017). For example, GRMZM2G359974 were significant associated with kernel width and weight in this study and a SNP which located on 0.9 Mb upstream of this gene were also found significantly associated with kernel width by GWAS (Liu et al., 2017), and a SNP associated with kernel width was located on 1.5 Mb upstream of GRMZM2G083894. GRMZM2G083894 were located in the QTL interval which is found by using the RIL population from the cross of B73 and BY804 (Liu et al., 2017). Based on these results, these three candidate genes should be considered the most promising candidates related to kernel size and weight in maize for further functional validation.

## Conclusion

Grain yield and related traits are complex quantitative traits controlled by numerous QTLs of small effect in maize. The integration and meta-analysis of a large set of QTLs provides valuable information for QTL fine mapping and key genes for cloning. In this study, we collected 999 QTLs related to yield and related traits and identified 76 MQTLs across the maize genome. Based on a comparative genomic strategy, several maize orthologs of rice yield-related genes were identified in the MQTL regions. We then mined the candidate genes or loci for kernel size and weight through regional association mapping based on the results of the meta-analysis. Consequently, three potential candidate genes associated with kernel size and weight within three MQTL regions were identified in this study. These results confirmed that combining meta-analysis and regional association mapping is helpful for functional marker development and rapid determination of candidate genes or loci, and the candidate loci identified in this study contribute to our understanding of the genetic architecture of grain yield and related traits in maize.

## Author contributions

TW and YuL: conceived and designed the study and carried out all the experiments; LC, YA, YoL, CL, DZ, YuS, and YaS: perform the field experiment and the evaluation of the phenotype: LC, YA, YoL, and CL: carried out the analysis. LC and YA: wrote the manuscript. All authors have read and approved the final version of the manuscript.

### Conflict of interest statement

The authors declare that the research was conducted in the absence of any commercial or financial relationships that could be construed as a potential conflict of interest.

## Supplementary material

Figure S1 Expression levels of candidate genes identified through regional association mapping during the different stages of maize kernel development.

Table S1 QTL information collected in this study.

Table S2 Annotated transcripts located in MQTL regions.

Table S3 Genes related to grain yield and associated traits in rice and homologous genes in maize.

Table S4 Comparison of meta-analysis results between this study and previous studies (Wang et al., 2013, 2016; Martinez et al., 2016).

Table S5 The MQTL region location based on B73 ref V4.

Table S6 Compared the positive allele between the linkage and association population.
