# Supplementary figures and images for: Candidate Loci for Yield-Related Traits in Maize Revealed by a Combination of MetaQTL Analysis and Regional Association Mapping
Source: Front Plant Sci. 2017 Dec 22;8:2190. doi: 10.3389/fpls.2017.02190 (PMC5744402; doi:10.3389/fpls.2017.02190)

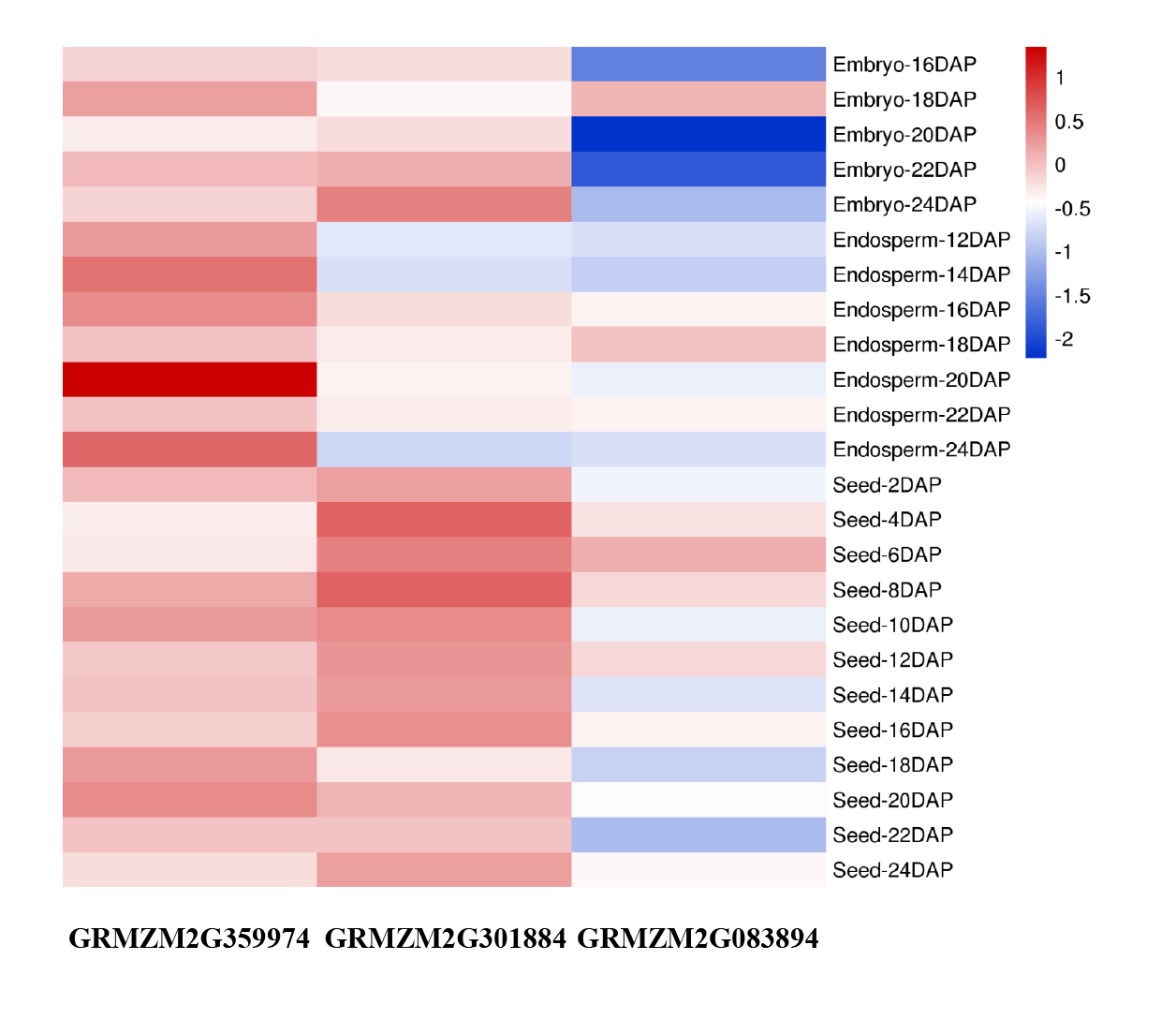

Supplement: Figure S1 — Expression levels of candidate genes identified through regional association mapping during the different stages of maize kernel development. [file Image1.TIF]
